# The Book World of Medicine and Science

**Published:** 1893-12-30

**Authors:** 


### The Book World of Medicine and Science.

A Text-book of General Therapeutics. By W. Hale White, M.D., F.R.C.P. (London: Macmillan and Co. 1889.)

This volume, as the preface informs us, represents a portion of the course of lectures delivered by the author to the students of Guy's Hospital—that portion devoted to the treatment of medical cases by means other than the administration of drugs. Without at all underrating the value of the drug treatment of disease, we cannot fail to notice how greatly the attitude of the practitioner towards his patient has changed as to this in modern days. The management of a case of enteric fever at the present date, as contrasted with its treatment some fifty years ago, is a good illustration of our meaning, the experienced physician of to-day regarding the selection and administration of drugs which received so much attention from his predecessors as matters altogether subordinate to the far more important considerations of suitable feeding, ventilation, cold bathing, and disinfection. Writing in The Hospital, it is our duty to point out to our readers that this change in the practice of the physician has greatly multiplied the duties, while it has, at the same time, greatly increased the usefulness and responsibilities of the nurse. The days when the nurse was mainly concerned with the dosing of the patient at stated intervals are now long past; her aims are now much higher than that, and her knowledge of the treatment of disease by means other than the administration of drugs can scarcely be too greatly extended; for this reason, books such as the one we are now reviewing are valuable additions to the nurses' library. The separate discussion of this branch of therapeutics is a good idea, and, we believe, original; at least, we know of no text-book yet published in which the outlines of modern views and modern methods are so fully and so concisely represented.

Dr. Hale White devotes his first chapters to climatology; but while admitting that he has in these collected much interesting and useful information, we cannot help thinking that too many details are given if the text-book is intended for the use of students only, and too few to entitle the book to be classed among works of reference on this subject for practitioners. The descriptions given of treatment by compressed air, lavage, Professor Certel's management of chronic cardiac disease, and the Weir-Mitchell system employed in the treatment of neurosis, are all clearly given, and the sound views held by the author are shown in the commentaries made on each. In the chapter on Massage it would perhaps have been better to have avoided all reference to the preliminary greasing of the skin; this practice is, we are convinced, always a mistake, and Dr. Hale White, as he tells us, has himself given it up.

The chapters on Diet and Baths are among the best in the book, though here and there we come upon sentences which need some modification. In discussing the conditions benefited by non-medicated cool baths, we are told that "an instance of the good gained by going to a hydropathic establishment is seen in the visits of Darwin to Ilkley and Moore Park." Of course, it is the benefit which Mr. Darwin derived from these visits that is meant, but even with this addition the statement is somewhat insufficient. In the last paragraph of Chapter VII. the distinction between the conditions known under the names of "rickets" and "acute rickets" is not made sufficiently apparent, although the cauliflower and orange juice given in the dietary recommended for the latter affection points to its recognition as a variety of scurvy, which we believe it really is. Electricity is treated of at considerable length, but very full details of the practical management of the agent can scarcely be expected in a text-book which deals with so many subjects. We notice one error in the description of the electrolysis of superfluous hairs which might lead to a rather unpleasant result if steel needles were used. The needle should always be connected with the kathode (not the anode); the positive pole (anode) is best held in the patient's hand, the current made or broken by the patient under the surgeon's direction. A steel needle if attached to the anode deposits iron in the skin as a permanent black stain. The chapters on Hypnotism and Metallo-therapy state concisely the facts regarding these matters as far as they have been determined; regarding their application to the treatment of disease, Dr. Hale White expresses views in which we fully coincide. Not the least praiseworthy feature in this book is the bibliography with which each chapter ends. On special subjects any insufficiency of detail can be at once supplemented by reference to the carefully selected original works, whose titles are given.

Sanitary Engineering and Architecture. Transactions of the Sanitary Institute. Vol. XIII. 1892. (Edward Stanford.)

The section of engineering and architecture met under the presidency of Mr. James Lemon, Mayor of Southampton, who delivered an address on the principles of sewer construction, and gave some sound advice as to the most important points to attend to, from a sanitary standpoint, inselecting a dwelling house. We endorse his advice that every intending tenant should satisfy himself of the sanitary condition of a house before taking it, and it is not too much to expect that the owner should bear the expense of the necessary examination. More debatable, perhaps, are the views expressed in favour of the free ventilation of sewers by grid ventilators in view of the paper recently read before the British Medical Association by Dr. Sydney Davies, in which statistics seem to show that the introduction of grid ventilators coincided with a marked increase of diphtheria in Plumstead. Sir Charles Cameron also pointed out objections to the number of openings usually thought necessary for the ventilation of sewers. Mr. Henry Law described an apparatus for softening water on a large scale. The water containing bicarbonate of lime is treated with lime water, when the insoluble carbonate is formed, and may be removed by subsidence in suitable tanks, or removed by filters, which were described. The importance of such a process in districts in which the water is very hard is considerable both for hygienic and engineering reasons, as the salts of lime are apt to be deposited on the inner surfaces of boilers and hot-water pipes, leading to great inconvenience and often to accidents. The cost was stated at one farthing per thousand gallons of water in Southampton. Mr. W. R. Maguire contributed a paper on the prevention of typhoid fever, chiefly from the engineering point of view, which deserves attention from all those responsible for sanitary work. He shows the many ways in which this scourge may be favoured by faulty arrangement and imperfect workmanship in the laying of house drains, and suggests that the only way to ensure proper attention to these matters is the appointment of a Minister of Health, with powers to compel individuals and corporations to do what is necessary for the health of the community. It is quite certain that typhoid fever exists in many places to an extent which is far from creditable to the authorities in view of the state of our knowledge of its cause and mode of dissemination. A study of Mr. Maguire's paper will indicate the directions in which defects are to be sought and suggest appropriate remedies. In a paper on soil pipe ventilation by Mr. H. R. Kenwood, it is stated that an investigation of the talc valve in common use shows it to be extremely unsatisfactory, as in many cases the current is reversed, and they serve as outlets instead of inlets. He makes the suggestion that the ventilating shaft should be surrounded by a spiral turn of hot-water pipe from the bath supply (carried outside and protected from frost by felt) so as to cause an exhaust action in the ventilator. This simple expedient was stated to have been successful in one instance in which it had been tried, but some doubt was cast on its practical value by those who took part in the discussion.

